# Embedding Postoperative Driving Advice Into Electronic Discharge Documentation After Elective Joint Replacement: A Closed-Loop Quality Improvement Project

**DOI:** 10.7759/cureus.118977

**Published:** 2026-11-02

**Authors:** Rishab Notaney, Rishi Notaney, Vinaen S Charadva, Michael Goldring

**Affiliations:** 1 Orthopaedics, University Hospitals of Leicester NHS Trust, Leicester, GBR; 2 Paediatrics, University Hospitals of Leicester NHS Trust, Leicester, GBR; 3 Medicine/Surgery, University Hospitals of Leicester NHS Trust, Leicester, GBR; 4 Shoulder and Elbow Orthopaedic Surgery, University Hospitals of Leicester NHS Trust, Leicester, GBR

**Keywords:** arthroplasty, discharge documentation, driving advice, dvla guidelines, patient safety, patient understanding, quality improvement projects

## Abstract

Background and objectives

Return to driving is an important consideration following joint replacement, with implications for patient safety, independence, and medico-legal responsibility. However, the consistency with which postoperative driving, Driver and Vehicle Licensing Agency (DVLA), and insurance advice is communicated, documented, and understood is unclear. This study evaluated the documentation and provision of postoperative driving, DVLA, and insurance advice following elective joint replacement, alongside patients’ awareness and junior doctors’ knowledge and confidence. The primary aim was to assess and improve the documentation of postoperative driving advice following elective total hip replacement (THR), total knee replacement (TKR), or total shoulder replacement (TSR). Secondary aims were to assess patient awareness of DVLA requirements and to explore junior doctors’ knowledge and confidence in providing postoperative driving, DVLA, and insurance advice.

Methods

A two-cycle, closed-loop quality improvement project was undertaken within the orthopaedic department at a tertiary teaching hospital. Baseline discharge documentation was reviewed for patients who underwent elective THR, TKR, or TSR between December 2025 and February 2026. Eligible patients were surveyed regarding receipt of postoperative driving advice and awareness of DVLA requirements. Junior doctors within the department completed a structured questionnaire assessing their knowledge and confidence in providing advice. A guideline-informed driving, DVLA, and insurance advice section was subsequently embedded within the discharge-summary template. A re-audit was undertaken in patients who underwent elective arthroplasty between March and April 2026, with repeat assessment of discharge documentation, eligible patient surveys, and junior doctor surveys in a new cohort of patients and junior doctors.

Results

Driving advice was documented in 0/101 baseline discharge summaries versus 63/78 (80.8%) post-intervention, an absolute increase of 80.8 percentage points (95% confidence interval (CI): 70.0-88.0; p < 0.001). Advice meeting predefined content criteria was documented in 62/78 (79.5%) post-intervention summaries vs. 0/101 at baseline (absolute increase 79.5 percentage points, 95% CI: 68.6-87.0; p < 0.001). The composite of documented or patient-reported advice increased from 30/63 (47.6%) to 32/34 (94.1%; p < 0.001). DVLA awareness was recorded for 15/61 (24.6%) baseline and 15/34 (44.1%) post-intervention respondents; evidence of a change was inconclusive (p = 0.066). Clinician findings were descriptive. The designated DVLA/insurance response was selected by 3/6 (50%) baseline and 4/6 (67%) post-intervention junior doctors, while mean confidence scores increased from 2.67 to 3.33 out of 5.

Conclusions

Embedding guideline-informed postoperative driving, DVLA and insurance advice within the electronic discharge template was associated with increased documentation of driving advice meeting predefined content criteria and a higher composite of documented or patient-reported advice. The intervention was simple and required no additional educational programme; it was designed to reduce reliance on individual clinician memory. Changes in patient DVLA notification awareness were inconclusive, and clinician findings were exploratory. Documentation did not establish patient receipt or comprehension. Sustainability beyond the two-month follow-up requires evaluation.

## Introduction

Returning to normal activities is an important component of recovery after joint replacement. For many patients, driving is essential for independence, employment, and social participation. However, postoperative impairment due to pain, restricted mobility, reduced strength, and the effects of analgesic medication may temporarily affect a patient’s ability to control a vehicle safely. Clear advice on when and under what circumstances driving can be resumed is therefore a key component of postoperative discharge counselling. The National Institute for Health and Care Excellence (NICE) guideline NG157 recommends that patients undergoing elective total hip replacement (THR), total knee replacement (TKR), and total shoulder replacement (TSR) should receive information on return to usual activities, which explicitly includes driving [1].

The Driver and Vehicle Licensing Agency (DVLA) guidance advises patients to establish with their healthcare professionals when it is safe to resume driving, and states that they must notify the DVLA when the effects of a medical condition or surgery continue to impair driving for more than three months [2]. Failure to notify the DVLA can result in a fine of up to £1,000, with potential prosecution if an accident occurs as a result. Furthermore, the DVLA emphasises that “drivers have the legal responsibility to remain in control of a vehicle at all times” and “drivers must ensure they remain covered by insurance to drive after surgery” [3]. Appropriate postoperative discharge summaries should therefore address functional readiness to drive, including relevant DVLA and insurance responsibilities.

The Royal College of Surgeons of England (RCS) has provided postoperative recovery advice for patients following hip and knee replacements, including advice on returning to driving [4,5]. These guidelines explicitly state that “your insurance company should be informed about your operation”. The RCS also states that driving should be avoided for the first six weeks after both THR and TKR, but emphasises that return to driving should be determined by functional readiness rather than postoperative time alone. Furthermore, the RCS outlines a specific practical test to gauge readiness to drive, which involves attempting an emergency stop without engine ignition. For both THR and TKR, the RCS states that patients should consider driving again only when sudden, uninhibited pressure can be applied to execute an emergency stop safely [4,5]. For TSR, no equivalent national RCS guidance is available. Local guidance from the Leicester Shoulder Unit advises that patients may return to driving from six weeks postoperatively, provided they are able to control the vehicle safely [6]. Like the RCS guidance, this guidance emphasises functional readiness as an important determinant of return to driving.

Despite this, postoperative driving advice may be inconsistently communicated and documented. Previous quality improvement (QI) initiatives have identified deficiencies in post-surgery driving advice and have used interventions such as staff education, visual reminders, and discharge prompts to improve practice. Tahsin et al. [7], in a QI project undertaken following elective inguinal hernia repair, reported improved provision of correct driving advice after a multimodal educational intervention. However, less is known about the reliability and sustainability of driving advice following elective joint replacement, or whether improved documentation translates into improved patient comprehension.

Informal discussions with approximately 10 postoperative patients on the elective orthopaedic ward suggested that comprehension of postoperative driving and insurance requirements varied. This prompted a formal assessment of current practice. We hypothesised that reliance on individual clinicians to remember and provide driving advice contributed to variation in practice. Embedding guideline-informed advice within the existing electronic discharge workflow was therefore selected as a simple system-level intervention aimed at reducing reliance on individual memory.

The primary aim was to assess and improve documentation of postoperative driving advice following elective THR, TKR, and TSR. A local target of 100% documentation was set to operationalise the NICE NG157 recommendation to provide information about returning to driving, using discharge-summary documentation as a measurable process standard. However, NICE does not specifically mandate documentation within the discharge summary. Secondary aims were to assess patients’ awareness of DVLA requirements and the different routes through which patients received driving advice, and to explore junior doctors’ knowledge and confidence in providing postoperative driving, DVLA, and insurance advice.

## Materials and methods

Study design and setting 

A two-cycle, closed-loop clinical audit and QI project was undertaken within the elective orthopaedic service at a tertiary teaching hospital within the United Kingdom. The project evaluated the provision and documentation of postoperative driving advice following elective THR, TKR, and TSR. Baseline measurement included patients undergoing eligible procedures between December 2025 and February 2026. Following implementation of the intervention, a re-audit was undertaken among patients undergoing eligible procedures between March and April 2026. Reporting of this quality improvement project was guided by the Standards for QUality Improvement Reporting Excellence (SQUIRE 2.0) guidelines [8].

Governance and ethical considerations 

The project was prospectively registered with University Hospitals of Leicester NHS Trust as a Quality Assurance project (project number: 14961). Under local institutional governance, it was not classified as research, and formal NHS Research Ethics Committee approval was not required. Patient-identifiable information was used only where necessary for patient identification, contact, and data linkage. Direct identifiers were deleted once data collection and linkage were complete. Participation in the telephone survey was voluntary, and only patients who agreed to participate completed the questionnaire. This project did not receive any funding.

Baseline data collection 

Adults undergoing elective THR, TKR or TSR, including revision arthroplasty, during the specified audit periods were eligible. Bilateral procedures, staged arthroplasty, transfers and in-hospital deaths were excluded. Eligible admissions were identified from theatre records using the electronic theatre management system and cross-referenced with Nervecentre, the Trust’s electronic patient record system. For each eligible admission, the presence or absence of postoperative driving advice within the discharge summary was recorded. Documented advice was considered accurate if it included advice regarding informing insurance companies, DVLA notification requirements, and assessment of functional readiness to drive through the ability to safely perform an emergency-stop manoeuvre. These operational criteria were informed by DVLA guidance and RCS patient information [2-5]. Individual procedure categories, age, sex and length of stay were not recorded in the analysis dataset. 

Patient contact details were also obtained on Nervecentre. Patients were subsequently contacted by telephone to complete a structured postoperative survey. Where patients could not be reached on the initial attempt, a second telephone call was made on a different day, with a maximum of two contact attempts per patient. Data collectors were aware of each patient’s audit cycle when reviewing discharge summaries and conducting telephone surveys; assessments were not blinded. Survey eligibility was established by asking whether patients currently drove or held a valid driving licence. Eligible patients who agreed to participate were asked whether they had received a patient information leaflet containing postoperative driving advice and whether they had received verbal driving advice. Discharge and telephone-call dates were not recorded in the analysis dataset, preventing calculation of discharge-to-survey intervals. 

Patients reporting verbal advice were initially asked to describe what they had been told, followed by a closed-ended question about whether the advice included the ability to perform an emergency-stop manoeuvre safely. Verbal advice was classified as meeting the emergency-stop criterion if patients recalled this advice spontaneously or confirmed having received it following the closed-ended question. DVLA notification awareness was assessed using an open-ended question about notification after surgery, followed by a closed-ended question asking whether patients knew they should contact the DVLA if their ability to drive had been affected for more than three months after the surgery. This follow-up supplied the notification advice. Responses therefore reflected recorded awareness of the stated advice rather than independently demonstrated understanding. The verbatim patient questionnaire and assessment criteria are provided in Appendix 1.

Data collection was undertaken by seven data collectors. For each patient, the same collector assessed the discharge summary and administered the telephone survey. The discharge-summary assessments and the recorded questionnaire responses were not independently checked by a second reviewer. Survey responses were linked at the individual-patient level to the corresponding electronic discharge-summary records using Microsoft Excel. This enabled assessment of advice provision through the different routes (discharge summary, patient information leaflet (PIL), verbal advice) and DVLA notification awareness for each patient. Junior doctors working within the orthopaedic department were invited to complete a structured five-item questionnaire administered electronically using Google Forms. The questionnaire was not formally validated or piloted. The questionnaire assessed knowledge of postoperative driving advice, understanding of DVLA and insurance requirements, and self-reported confidence in counselling patients following elective joint replacement.

The full five-item junior doctor questionnaire is provided in Appendix 2. Responses were analysed separately for each item. For the timeframe questions, the proportions selecting six weeks after right THR and four to six weeks after right TKR were reported. The THR response was consistent with the general timeframe in RCS patient information [4]. The TKR response category did not precisely match the RCS guidance, which suggests assessment of fitness to drive after six weeks [5]. We therefore reported the proportion selecting ‘four to six weeks’ without classifying this response as definitively correct according to RCS guidance.

For the combined DVLA/insurance question, the fourth option, which specified informing the insurer and notifying the DVLA if unable to drive after three months, was the designated response. The DVLA component was informed by guidance on postoperative driving restrictions [2], while insurer notification was supported by RCS patient information [4,5]. Limitations of the question’s wording are explained in Appendix 2. Confidence was summarised using the mean and median (range), with higher scores indicating greater confidence. Free-text responses were classified according to whether they explicitly mentioned the ability to perform an emergency stop, including responses that also expressed uncertainty. Responses stating only “unsure” or omitting this criterion were classified as not identifying it.

Outcome measures 

Primary Outcome

Proportion of discharge summaries containing documented postoperative driving advice. 

Additional Documentation Outcome

Proportion of discharge summaries containing advice meeting the predefined accuracy criteria. 

Secondary Patient Outcomes

Receipt of verbal driving advice, accuracy of verbal advice, receipt of a PIL, provision of driving advice through at least one assessed route, and correct awareness of DVLA notification requirements. 

Provision of driving advice through at least one assessed route was defined as a composite of one or more of the following: patient-reported verbal advice meeting the predefined emergency-stop criterion, discharge-summary advice meeting the predefined accuracy criteria, or patient-reported receipt of a PIL containing postoperative driving advice. Each patient was counted once, irrespective of the number of qualifying routes. Patients routinely receive their discharge summary in accordance with the Trust’s published discharge information [9]. Hence, inclusion of driving advice in the letter was treated as documented provision of written advice for this composite. However, individual receipt, reading, and understanding were not assessed. PIL content and accuracy were not independently evaluated. The composite therefore combined documentation with patient-reported advice and did not establish verified receipt or comprehension across all routes. Its inclusion of discharge-summary documentation also means that it overlaps with the process directly targeted by the intervention.

An additional analysis undertaken in response to peer review excluded discharge-summary documentation from the composite, retaining patient-reported PIL receipt or verbal advice meeting the predefined emergency-stop criterion. Each patient was counted once, irrespective of whether one or both criteria were met. For composite outcomes, a positive result on any qualifying component established a positive composite despite missing data on another component. Where no component was positive and at least one was missing, composite status was treated as missing and excluded from that analysis.

The DVLA awareness outcome was the proportion of patient survey respondents recorded as aware of the notification advice stated in the survey item. Patient awareness was assessed only for DVLA notification, not insurance responsibilities or functional readiness.

Exploratory Clinician Measures

Expected THR and TKR return-to-driving timeframes, knowledge of DVLA/insurance requirements, recognition of key safe-driving criteria in open-text responses, and self-reported confidence. 

Theory of change and driver diagram 

At baseline, provision of postoperative driving advice depended largely on individual clinicians recognising the need for counselling, recalling the relevant guidance and documenting this information during discharge. We hypothesised that this reliance on individual clinician knowledge and memory contributed to inconsistent provision and documentation of advice. Our intervention was therefore to embed guideline-informed driving, DVLA, and insurance information within the existing electronic discharge-summary template. By integrating the advice into a routine step in the discharge workflow, we aimed to reduce reliance on individual clinician memory and improve the reliability and consistency of information provision. 

We hypothesised that more reliable documentation would increase patients' exposure to appropriate postoperative driving advice and that increased exposure might, in turn, improve awareness of DVLA requirements. Patient awareness of DVLA requirements was therefore measured (using patient surveys) separately from documentation to determine whether improved information provision was accompanied by improved awareness. The different routes through which patients received advice, including the discharge summary, verbal counselling and PILs, were also assessed to explore how the intervention affected the wider process of information provision. 

Junior doctors routinely received pre-existing induction teaching on use of the discharge-summary template; this teaching was unchanged during the project, and no additional intervention was introduced as part of the QI intervention. Maintaining the existing educational programme (i.e., only induction teaching) allowed the effect associated with the workflow intervention to be evaluated without introducing an additional planned educational change. Junior-doctor knowledge and confidence regarding postoperative driving, DVLA and insurance advice were nevertheless measured before and after implementation as exploratory outcomes to assess whether exposure to guideline-informed information within the discharge workflow was associated with changes in clinician knowledge or confidence. 

A driver diagram (Figure 1) was developed to represent this theory of change. Three primary drivers were identified: (1) reliable provision and documentation of driving advice, (2) patient awareness of DVLA requirements, and (3) junior-doctor knowledge and confidence. The intervention was expected to directly improve provision and documentation of advice, with potential downstream effects on patient understanding of DVLA requirements and junior-doctor knowledge and confidence.

**Figure 1:**
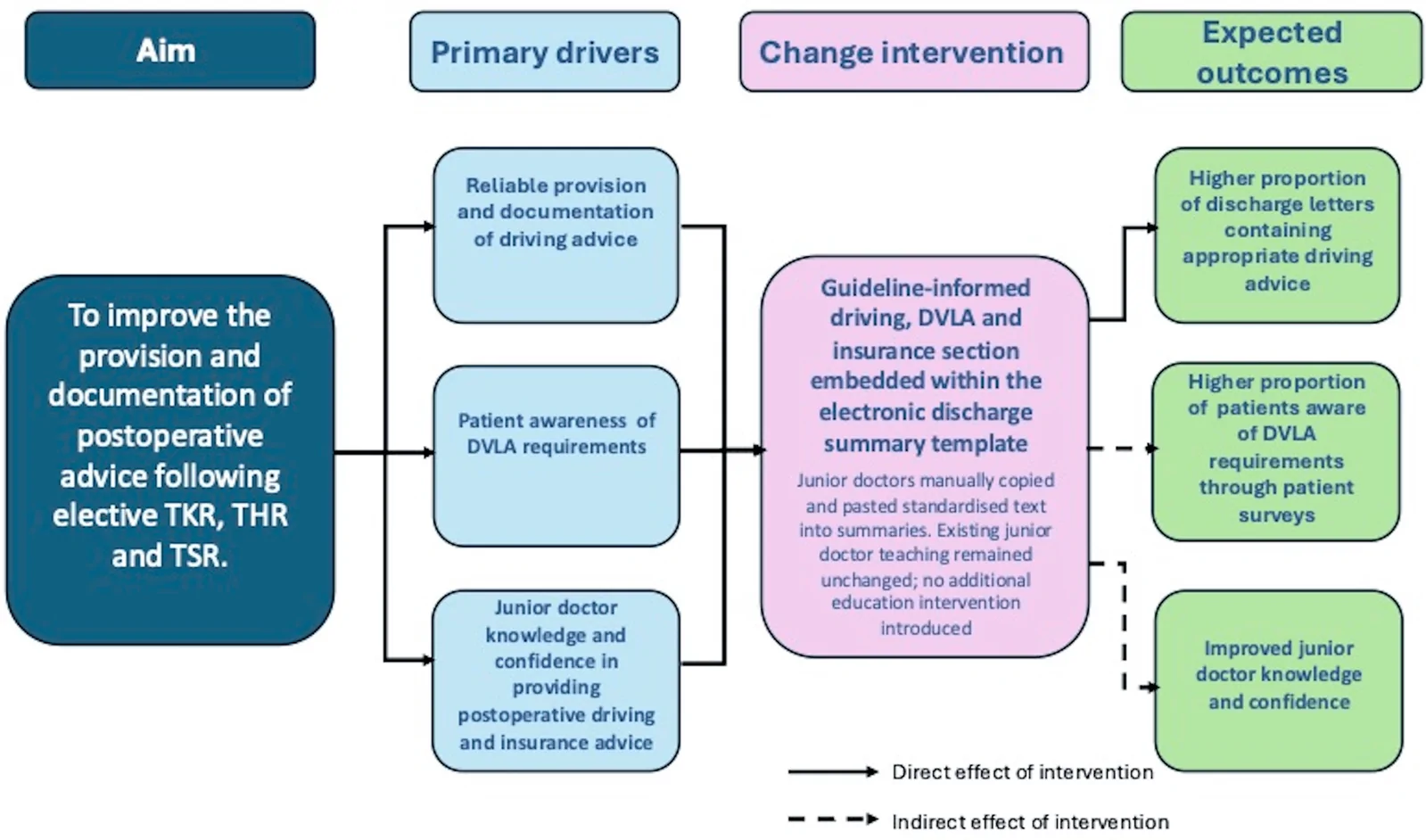
Driver diagram illustrating the theory of change for improving postoperative driving advice after elective joint arthroplasty TKR: total knee replacement; THR: total hip replacement; TSR: total shoulder replacement; DVLA: Driver and Vehicle Licensing Agency Image credit: Rishab Notaney (author)

Intervention 

Following baseline measurement and approval by the project-supervising consultant and departmental lead, a section containing guideline-informed postoperative driving, DVLA and insurance information was incorporated into the electronic discharge-summary template used by junior doctors (Figure 2). The section added advised patients to return to driving only once they had recovered enough to maintain safe control of the vehicle, including the ability to perform an emergency stop without limitation from pain or restricted mobility, consistent with RCS and local guidance [4-6]. The section also included relevant information regarding DVLA notification and insurance responsibilities. The template’s “typically six to eight weeks” wording was selected as a conservative general timeframe and approved by the supervising consultant (Michael Goldring, co-author). It was not intended to reproduce the cited six-week guidance for THR, TKR and TSR exactly or to indicate that elapsed time alone established fitness to drive. Return to driving remained dependent on individual recovery and the ability to control the vehicle safely. 

**Figure 2:**
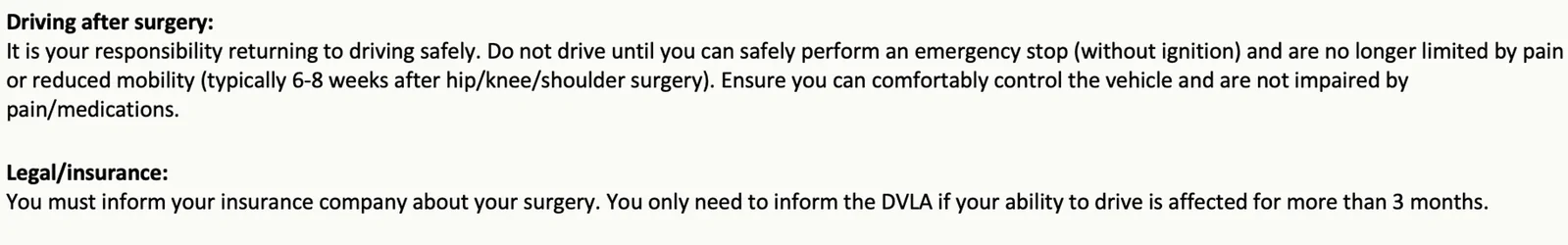
Guideline-informed postoperative driving, DVLA, and insurance advice incorporated into the electronic discharge-summary template DVLA: Driver and Vehicle Licensing Agency

The intervention was designed to incorporate driving advice into the routine discharge workflow rather than relying on individual clinicians to remember to write this information independently. No additional educational intervention was introduced during the implementation period, with existing junior doctor induction arrangements remaining unchanged. The standardised electronic template driving-advice text was available for clinicians to copy and paste manually into the electronic discharge summary; it was not pre-populated automatically. Clinicians could edit or delete the text. Inclusion therefore required an active insertion step, although its presence did not establish that the advice had been individually reviewed or discussed with the patient. 

Re-audit and clinician survey 

Following implementation of the intervention, re-audit was undertaken among a new cohort of patients undergoing eligible procedures between March and April 2026, using the same inclusion and exclusion criteria. This second audit cycle repeated the documentation measures and patient surveys. Post-intervention clinician surveys were completed by a new cohort of junior doctors following departmental rotation. Accordingly, pre- and post-intervention responses represented independent rather than paired samples. Given the expected small sample size in each cycle, junior doctor-reported confidence and knowledge were considered exploratory and were analysed descriptively without formal hypothesis testing. 

All eligible discharge records identified during each audit period were reviewed, and survey denominators reflect available responses from eligible participants for each outcome.

Statistical analysis 

Patients who could not be contacted, declined participation, or had no recorded survey responses were excluded from patient-survey analyses, while their discharge-summary data remained eligible for the documentation analysis. Available responses from partially completed surveys were retained. Missing or uninterpretable responses were excluded from the denominator for the corresponding outcome and were not coded as negative responses. No missing values were imputed. Denominators were therefore outcome-specific. For verbal-advice accuracy, a confirmed absence of verbal advice was classified as not having received accurate verbal advice; an unanswered accuracy question following reported receipt of verbal advice was treated as missing. 

Categorical variables were summarised as frequencies and percentages. Wilson 95% confidence intervals (CI) were calculated for individual proportions. Absolute differences were calculated as post-intervention minus baseline proportions and expressed in percentage points, with 95% CIs calculated using the Newcombe-Wilson method without continuity correction. Two-sided Fisher's exact tests were used for categorical comparisons. P < 0.05 was considered statistically significant.

Junior doctor categorical responses were summarised as counts with whole-number percentages. Confidence scores were summarised using both mean and median (range); these summaries were descriptive. As clinician samples were small and independent, these outcomes were considered exploratory, and no formal hypothesis testing was performed for clinician outcomes. 

## Results

Discharge documentation 

A total of 101 eligible discharge summaries were reviewed at baseline and 78 following implementation of the intervention. At baseline, postoperative driving advice was documented in 0 out of 101 discharge summaries (0.0%, 95% CI: 0.0-3.7%). Following implementation, driving advice was documented in 63 out of 78 discharge summaries (80.8%, 95% CI: 70.7-88.0%), representing an absolute increase of 80.8 percentage points (95% CI: 70.0-88.0; p < 0.001). Advice meeting the predefined accuracy criteria was documented in 62 out of 78 post-intervention discharge summaries (79.5%, 95% CI: 69.2-87.0%), compared with 0/101 at baseline, representing an absolute increase of 79.5 percentage points (95% CI: 68.6-87.0; p < 0.001). Among post-intervention summaries containing any driving advice, 62 out of 63 (98.4%) met the predefined accuracy criteria (Table 1; Figure 3).

**Table 1:** Documentation of postoperative driving advice before and after intervention Absolute differences are expressed in percentage points CI: confidence interval; pp: percentage points

Outcome	Baseline, n/N (%; 95% CI)	Post-intervention, n/N (%; 95% CI)	Absolute difference, pp (95% CI)	P-value
Driving advice documented	0/101 (0.0%; 0.0–3.7)	63/78 (80.8%; 70.7–88.0)	+80.8 (70.0 to 88.0)	< 0.001
Accurate driving advice documented	0/101 (0.0%; 0.0–3.7)	62/78 (79.5%; 69.2–87.0)	+79.5 (68.6 to 87.0)	< 0.001

**Figure 3:**
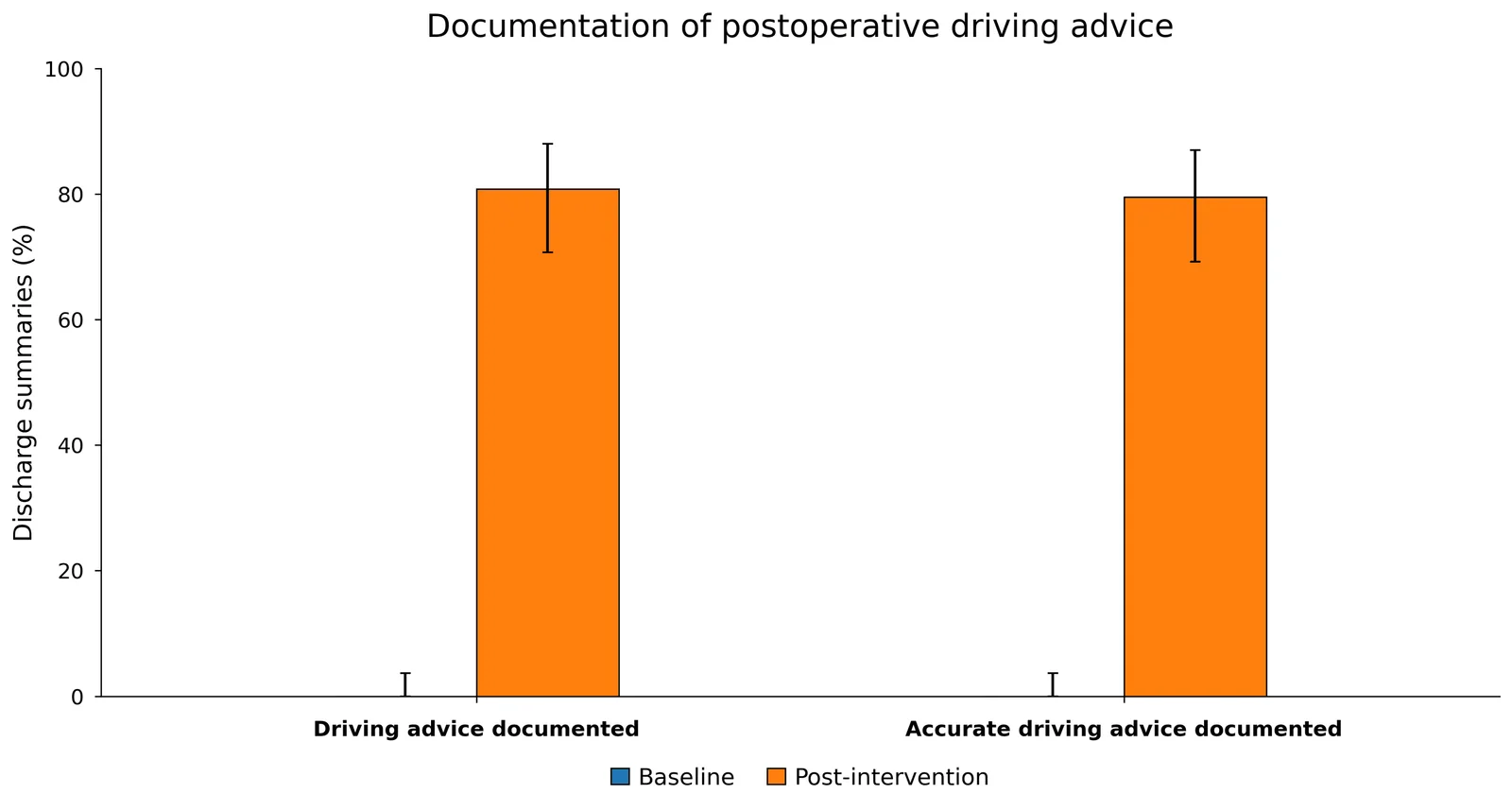
Documentation of postoperative driving advice before and after the intervention Bars show the proportion of discharge summaries containing any postoperative driving advice (left) and advice meeting predefined accuracy criteria (right), before and after the intervention. Error bars represent 95% confidence intervals for each proportion

Patient survey and linked documentation outcomes 

At baseline, 81 of 101 patients were successfully contacted, 19 could not be contacted, and one was recorded as deceased. Of those contacted, 63 eligible patients participated, 17 were ineligible, and one declined. Post-intervention, 54 of 78 patients were successfully contacted, 21 could not be contacted, and contact outcomes were unrecorded for three. Of those contacted, 34 eligible patients participated, 19 were ineligible, and one declined. Eligibility was not documented for the patient who declined in each cycle. Figure 4 summarises contact outcomes, eligibility and survey participation in both cycles.

**Figure 4:**
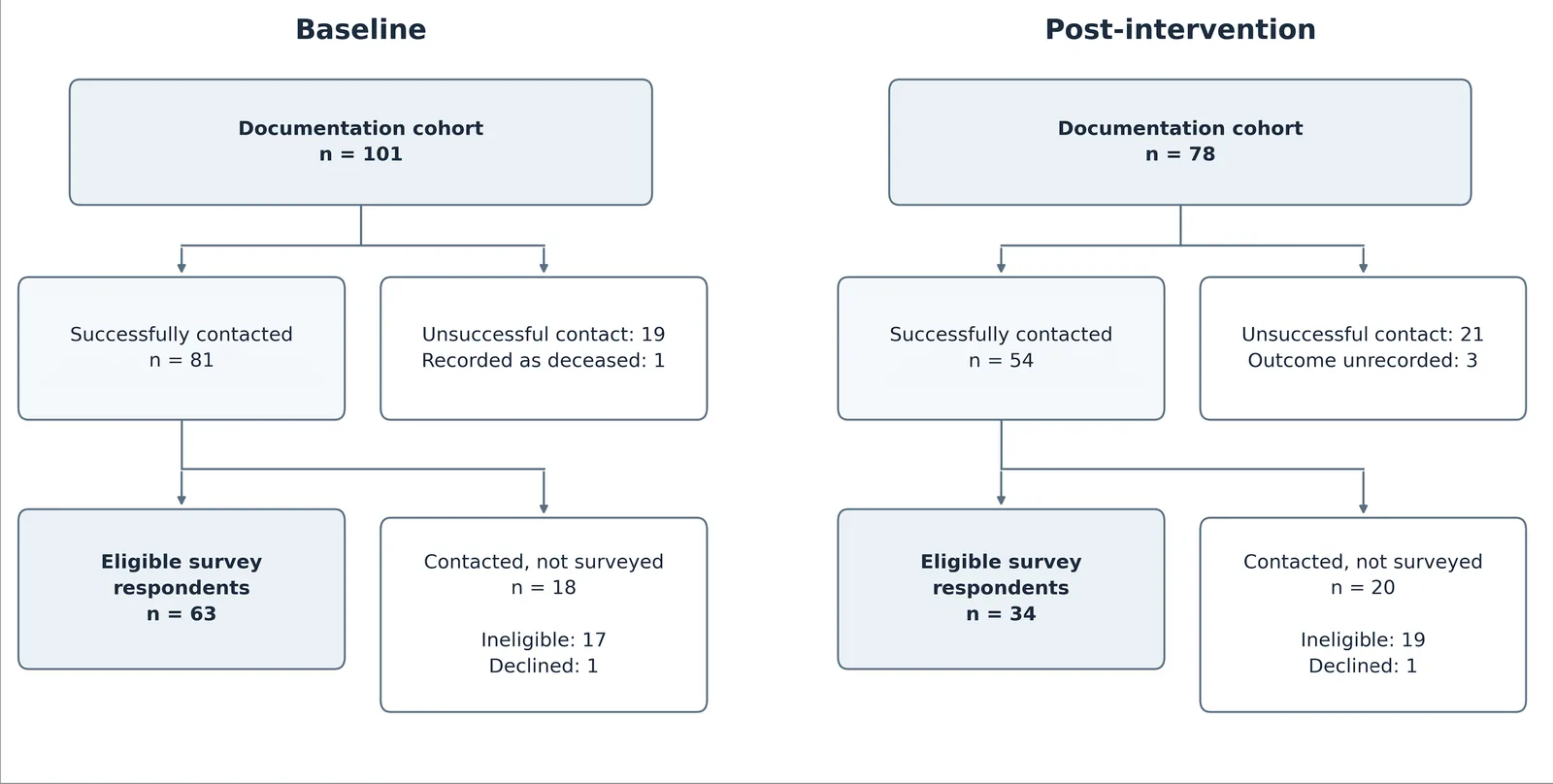
Participant flow through telephone contact, eligibility assessment, and survey participation before and after the intervention All patients in the initial cohorts contributed to the discharge-documentation analysis. Telephone-survey participation was restricted to patients who currently drove or held a valid driving licence and agreed to participate. Survey eligibility was not documented for the patient who declined participation in each cycle. The baseline patient recorded as deceased is shown as a contact outcome, not an in-hospital death. Outcome-specific survey denominators vary because missing or uninterpretable responses were excluded from the corresponding analyses

The proportion of surveyed patients meeting the composite criterion for advice provision through at least one assessed route increased from 30 out of 63 (47.6%, 95% CI: 35.8-59.7%) at baseline to 32 out of 34 (94.1%, 95% CI: 80.9-98.4%) at post-intervention. This represented an absolute increase of 46.5 percentage points (95% CI: 28.6 to 59.1; p < 0.001). When discharge-summary documentation was excluded, the proportion reporting PIL receipt or verbal advice meeting the predefined emergency-stop criterion was 30/63 (47.6%) at baseline and 14/33 (42.4%) post-intervention. The absolute difference was −5.2 percentage points (95% CI: −24.6 to 15.3; p = 0.671), providing inconclusive evidence of a change in coverage through these patient-reported routes.

Awareness of DVLA notification requirements increased from 15 out of 61 patients (24.6%, 95% CI: 15.5-36.7%) to 15 out of 34 patients (44.1%, 95% CI: 28.9-60.5%), corresponding to an absolute increase of 19.5 percentage points (95% CI: 0.1 to 38.3; p = 0.066). The Newcombe-Wilson confidence interval narrowly excluded zero, whereas the two-sided Fisher’s exact test did not meet the prespecified significance threshold (p = 0.066). These procedures use different statistical approaches and are not equivalent inversions of one another, so their conclusions can differ near the significance threshold. Evidence of a change in DVLA notification awareness was therefore considered inconclusive.

For the individual routes of advice, patient-reported receipt of a PIL was 17/63 (27.0%) at baseline and 7/33 (21.2%) post-intervention. The receipt of verbal advice was 27/63 (42.9%) and 12/34 (35.3%), respectively. Accurate verbal advice was reported by 15/63 (23.8%) patients at baseline and 10/34 (29.4%) post-intervention (Table 2). 

**Table 2:** Advice provision and DVLA notification awareness among surveyed patients ^*^Additional analysis undertaken in response to peer review, excluding discharge-summary documentation from the original composite. Patients qualified if they reported receiving a PIL containing driving advice or verbal advice meeting the predefined emergency-stop criterion; each patient was counted once. One post-intervention respondent had missing PIL data and no positive qualifying verbal-advice response and was therefore excluded from the composite excluding documentation. They remained positive for the original composite because their discharge summary contained advice meeting the predefined accuracy criteria CIs for individual proportions are Wilson 95% CIs. Absolute differences are reported as PP changes with 95% CIs. Denominators vary by outcome because missing or uninterpretable responses were excluded from the corresponding analysis DVLA: Driver and Vehicle Licensing Agency; CI: confidence interval; pp: percentage points

Outcome	Baseline, n/N (%; 95% CI)	Post-intervention, n/N (%; 95% CI)	Absolute difference, pp (95% CI)	P-value
Provision of driving advice through ≥ 1 assessed route	30/63 (47.6%; 35.8–59.7)	32/34 (94.1%; 80.9–98.4)	+46.5 (28.6 to 59.1)	< 0.001
PIL receipt or verbal advice meeting the predefined criterion^*^	30/63 (47.6%; 35.8–59.7)	14/33 (42.4%; 27.2–59.2)	−5.2 (−24.6 to 15.3)	0.671
DVLA notification awareness	15/61 (24.6%; 15.5–36.7)	15/34 (44.1%; 28.9–60.5)	+19.5 (0.1 to 38.3)	0.066
PIL received	17/63 (27.0%; 17.6–39.0)	7/33 (21.2%; 10.7–37.8)	−5.8 (−21.8 to 13.3)	0.625
Verbal advice received	27/63 (42.9%; 31.4–55.1)	12/34 (35.3%; 21.5–52.1)	−7.6 (−26.0 to 12.8)	0.520
Accurate verbal advice	15/63 (23.8%; 15.0–35.6)	10/34 (29.4%; 16.8–46.2)	+5.6 (−11.7 to 24.5)	0.629

Figure 5 illustrates the proportions of surveyed patients with evidence of driving advice through at least one assessed route and awareness of DVLA notification requirements, before and after the intervention.

**Figure 5:**
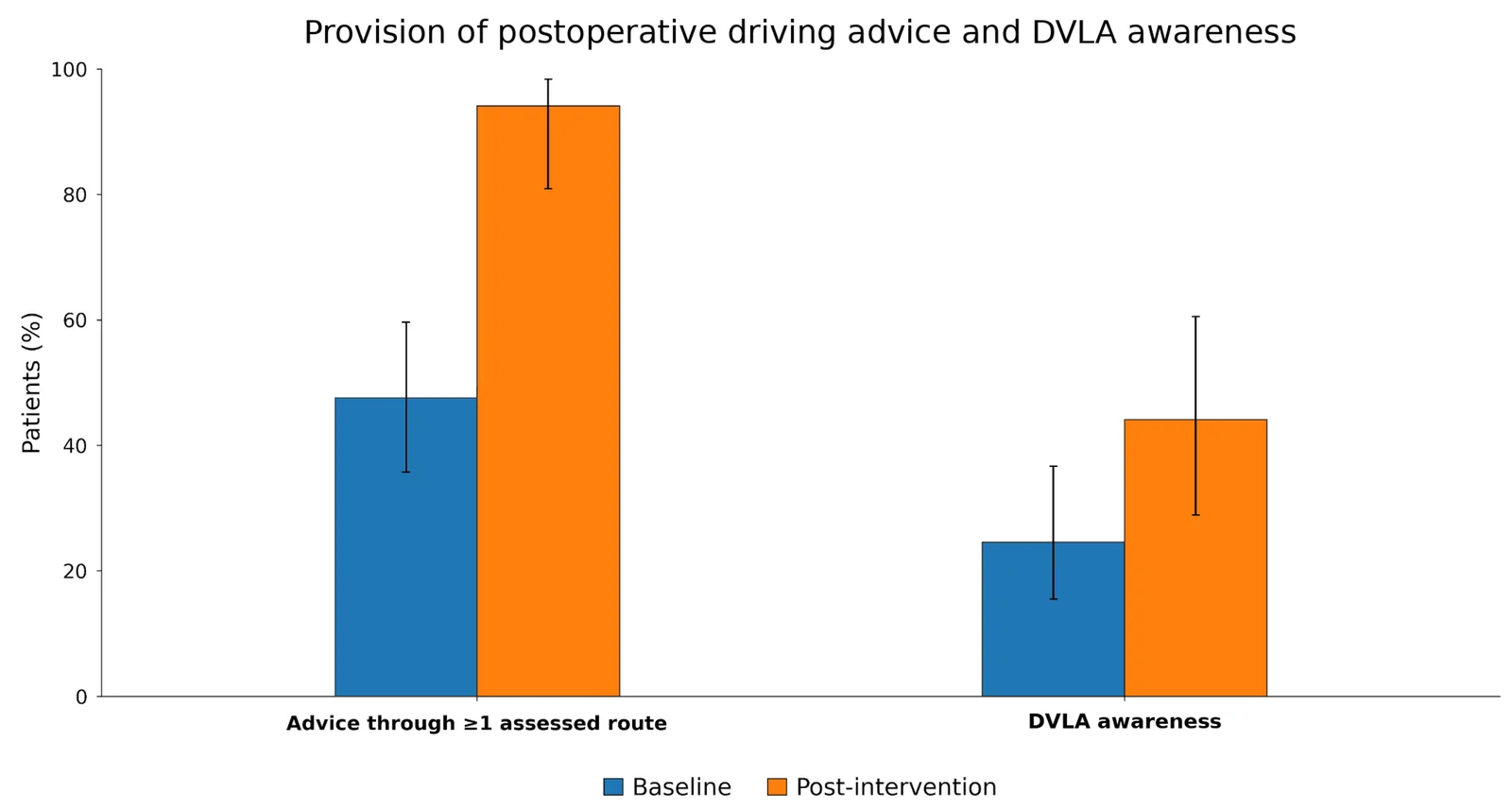
Provision of postoperative driving advice through at least one assessed route and awareness of DVLA notification requirements before and after intervention Error bars represent 95% CIs around individual proportions DVLA: Driver and Vehicle Licensing Agency; CI: confidence interval

Junior doctor knowledge and confidence 

Six junior doctors completed the baseline survey before the intervention was implemented. Following departmental rotation, a new cohort of six junior doctors completed the same survey after the intervention was implemented. For the expected return-to-driving timeframe following right THR, three out of six (50%) baseline respondents selected six weeks compared with six out of six (100%) post-intervention. For right TKR, three out of six (50%) selected four to six weeks at baseline compared with four out of six (67%) post-intervention; the remaining two post-intervention respondents selected 8-12 weeks. The designated DVLA/insurance response was selected by three out of six (50%) baseline respondents and four out of six (67%) post-intervention respondents. Mean self-reported confidence in providing postoperative driving advice increased from 2.67/5 to 3.33/5. Median confidence increased from 2.5/5 (range: 2-4) to 3.0/5 (range: 2-5).

Baseline open-text responses demonstrated variation in knowledge of functional criteria for safe return to driving. Three of six respondents explicitly referred to the ability to perform an emergency stop; two responded with only 'unsure', and one highlighted medication-related impairment and patient responsibility. After intervention implementation, four of six respondents explicitly identified the ability to perform an emergency stop as a key consideration, while two responded with only 'unsure'. Given the small, independent clinician samples, these outcomes were considered exploratory and are presented descriptively without formal hypothesis testing (Table 3). 

**Table 3:** Survey-assessed junior doctor knowledge and confidence regarding postoperative driving advice before and after intervention Values are n/N (%) unless otherwise stated. Confidence was rated on a 5-point scale THR: total hip replacement; TKR: total knee replacement; DVLA: Driver and Vehicle Licensing Agency

Outcome	Baseline (n = 6), n/N (%)	Post-intervention (n = 6), n/N (%)
Selected 6 weeks following right THR	3/6 (50%)	6/6 (100%)
Selected 4–6 weeks following right TKR	3/6 (50%)	4/6 (67%)
DVLA/insurance knowledge: designated response selected	3/6 (50%)	4/6 (67%)
Confidence, mean, /5	2.67	3.33
Confidence, median (range), /5	2.5 (2–4)	3.0 (2–5)
Open-text response mentioning emergency-stop ability	3/6 (50%)	4/6 (67%)

## Discussion

Principal findings 

This closed-loop QI project identified a substantial gap between national recommendations and routine postoperative discharge practice following elective joint replacement. Embedding guideline-informed driving, DVLA, and insurance information within the electronic discharge workflow was associated with a marked improvement in the primary process outcome, with documentation of postoperative driving advice increasing from 0% to 80.8%. Importantly, this improvement was accompanied by a high level of accuracy: 62 of the 63 (98.4%) post-intervention discharge summaries containing driving advice met the predefined accuracy criteria, corresponding to 79.5% of all post-intervention discharge summaries. Because the accuracy criteria mirrored the template content, this finding primarily reflects inclusion of standardised advice rather than independent clinician judgement or individualised counselling.

The proportion of surveyed patients meeting the composite criterion for documented or patient-reported advice increased from 47.6% to 94.1%. However, this increase was driven by discharge-summary documentation; the additional composite excluding this route did not demonstrate improved coverage through other qualifying patient-reported routes. DVLA awareness was numerically higher after implementation (24.6% versus 44.1%), but evidence of a change was inconclusive. Junior doctor findings were descriptive and exploratory because of the small, independent samples.

Documentation does not necessarily translate into awareness

An important finding was the distinction between documented or patient-reported advice provision and DVLA awareness. Following implementation, 94.1% of surveyed patients met the composite advice-provision criterion, whereas 44.1% met the survey criterion for DVLA awareness. The increase in the original composite was driven by discharge-summary documentation, the outcome directly targeted by the intervention. The additional analysis excluding this route did not demonstrate improved coverage through patient-reported routes (verbal or PILs). Discharge-summary documentation alone does not establish that advice reached the patient or was read, understood or retained. Patients routinely receive their discharge summary in accordance with the Trust’s published discharge information [9]. However, individual receipt, reading and comprehension of the driving advice were not verified in this project and should be assessed in future improvement cycles.

DVLA awareness was numerically higher after the intervention, but evidence of a change was inconclusive. The Newcombe-Wilson 95% confidence interval for the difference narrowly excluded zero (0.1-38.3 percentage points), whereas the two-sided Fisher’s exact test did not meet the prespecified significance threshold (p = 0.066). Furthermore, the survey combined spontaneous knowledge with prompted recognition and did not independently establish comprehension. These findings support evaluating patient awareness and comprehension alongside documentation in future improvement cycles.

Interpretation in relation to theory of change 

The findings are consistent with the proposed mechanism of the intervention (Figure 1). Before implementation, provision of driving advice depended on individual clinicians recognising the need for counselling, recalling the relevant guidance, and documenting it during discharge. Embedding guideline-informed advice within the electronic discharge workflow was intended to reduce reliance on individual memory and make documentation more consistent. This is particularly relevant in departments with frequent junior doctor rotation, where clinician-dependent processes may be vulnerable to staff turnover. The largest observed improvement concerned documentation, the process directly targeted by the intervention. Changes in patient awareness were inconclusive, and clinician findings were exploratory. The findings therefore support further evaluation of workflow standardisation alongside educational and communication strategies.

Comparison with previous QI work 

Tahsin et al. evaluated postoperative driving advice following elective inguinal hernia repair within the same NHS Trust. Their multimodal intervention incorporated staff education, visual and email reminders and was associated with an increase in discharge letters containing correct driving advice from 14.3% to 42.5%, alongside improved clinician knowledge [7]. Similarly, Buss and Gould identified an improvement in documented driving advice following abdominal surgery from 0% to 75% following sequential educational interventions. The final cycle also coincided with the discharge summary’s patient-advice section becoming compulsory [10]. Their findings therefore highlight the potential contribution of both education and changes to electronic documentation. 

In the present project, embedding guideline-informed advice within the discharge template, without additional education, was associated with an increase in documentation from 0% to 80.8%. Differences in populations, outcome measures, and interventions limit direct comparison. Nevertheless, these studies suggest complementary roles for education and workflow standardisation in supporting clinician knowledge and reliable documentation. Education may strengthen clinicians' awareness and ability to counsel patients, whereas workflow standardisation may improve the reliability with which information is delivered and documented. A combination of these approaches may therefore be more effective than either strategy alone. The present project also extends previous work by assessing patient awareness rather than documentation alone. This identified a residual communication gap, which supports evaluating combined educational and workflow interventions in future improvement cycles. 

Individual communication routes and unintended consequences 

Patient-reported PIL receipt was numerically lower post-intervention (27.0% versus 21.2%), as was receipt of any verbal advice (42.9% versus 35.3%). In contrast, reported verbal advice meeting the predefined emergency-stop criterion was numerically higher (23.8% versus 29.4%). When discharge-summary documentation was excluded from the composite, combined coverage through PIL receipt or verbal advice meeting this criterion was 47.6% at baseline and 42.4% post-intervention (difference: −5.2 percentage points, 95% CI: −24.6 to 15.3; p = 0.671). These estimates were imprecise and did not establish changes in patient-reported coverage or demonstrate that the intervention reduced counselling or leaflet provision (Table 2).

The project was not designed to determine why these patterns occurred. One possible explanation is that clinicians increasingly relied on the standardised discharge-summary template once an accessible written source of information became routinely available. If so, this would represent a potential unintended consequence of system standardisation: improved electronic provision may substitute for, rather than complement, direct communication. This interpretation remains speculative, but it provides an important learning point for the next improvement cycle. A robust discharge process should ideally combine reliable written documentation with consistent verbal reinforcement and patient-facing information rather than depend on a single communication route. A future cycle could therefore evaluate brief teach-back alongside written advice. A randomised trial in patients undergoing lumbar disc surgery found that teach-back discharge education improved patient discharge-education satisfaction, knowledge and discharge readiness [11]. 

Junior doctor knowledge and confidence 

Junior doctor findings were descriptive and exploratory. The designated DVLA/insurance response was selected by three out of six (50%) baseline respondents and four out of six (67%) post-intervention respondents. Mean confidence scores were 2.7 and 3.3 out of 5, respectively, with corresponding medians of 2.5 and 3.0. Selection of six weeks following right THR was recorded for three out of six (50%) and six out of six (100%), while emergency-stop ability was mentioned in open-text responses by three out of six (50%) and four out of six (67%).

Due to a limited number of junior doctors in the department, only six junior doctors participated in each cycle, and the post-intervention group represented a new cohort following departmental rotation, precluding paired analysis. The small, independent samples and questionnaire wording limitations described in Appendix 2 prevent firm conclusions about an educational effect of the intervention. Nevertheless, the findings are consistent with the possibility that embedding standardised guidance within routine clinical workflow may provide repeated passive reinforcement for clinicians as well as information for patients. This warrants further evaluation in a larger sample and supports combining workflow standardisation with recurring education during junior-doctor induction. 

Strengths and limitations

This project has several strengths. It completed a closed audit cycle informed by national guidance and evaluated outcomes at system, patient, and clinician levels. Discharge documentation was assessed not only for the presence of driving advice but also against predefined accuracy criteria, allowing the intervention to be evaluated in terms of both documentation reliability and content accuracy. The intervention was simple and embedded within routine clinical practice, with an explicit theory of change informing its evaluation. Patient surveys complemented the documentation review by assessing reported receipt of advice and recorded DVLA awareness, providing information that discharge-summary assessment alone could not capture.

Several limitations should be acknowledged. The single-centre before-and-after QI design limits causal inference. Only aggregated results for each audit cycle were available for temporal analysis, preventing assessment of weekly or fortnightly variation. Without a concurrent control group, secular trends, seasonal effects and unmeasured co-interventions cannot be excluded. No balancing measures were prospectively defined. Patient-reported verbal-advice receipt was considered retrospectively as a potential balancing measure, although the imprecise estimate does not establish that the intervention reduced verbal counselling. Data collectors were aware of the audit cycle, introducing potential observer and interviewer bias in discharge-summary assessments and the elicitation and classification of survey responses.

Predefined criteria and structured questions supported consistency but did not eliminate this risk, and assessments were not independently checked. Hence, although no additional educational intervention was introduced as part of the project, the observed changes cannot be attributed solely to the discharge-template intervention. The project evaluated one principal change intervention followed by re-audit rather than multiple iterative plan-do-study-act (PDSA) cycles. The two-month post-intervention measurement period does not establish sustainability; persistence of the documentation improvement across subsequent staff rotations requires further evaluation. 

Patient surveys involved independent cohorts (63 versus 34 respondents) and were susceptible to recall and non-response bias. Discharge-to-survey intervals were not recorded and may have differed between cycles, introducing possible differential recall bias. The closed-ended verbal-advice question explicitly mentioned the emergency-stop criterion and may have encouraged affirmative responses; this outcome therefore reflects reported recollection rather than independently verified counselling. DVLA awareness combined correct open-ended responses with affirmative responses to a closed-ended follow-up that supplied the notification advice. The outcome therefore did not distinguish spontaneous knowledge from prompted recognition and could have overestimated pre-existing awareness in both baseline and post-intervention cohorts.

The original composite advice-provision outcome included discharge-summary documentation directly targeted by the intervention; its increase therefore does not independently demonstrate improved patient communication, and individual receipt, reading and comprehension of documented advice were not verified. Survey participation and the proportion of contacted patients found to be ineligible differed between cycles, while eligibility remained unknown for some non-participants. Procedure categories, age, sex and length of stay were not recorded, preventing assessment of cohort comparability or differences between respondents and non-participants. Differences in patient composition and participation may therefore have influenced survey findings. Wide confidence intervals limited the precision of secondary outcome estimates.

Junior doctor samples were also small and independent (six per cycle), so findings were exploratory. The TKR questionnaire offered a 4-6-week response category, which did not precisely align with the six-week assessment point described in the RCS guidance [5]. Responses were therefore reported descriptively and should not be interpreted as definitive evidence of guideline-concordant knowledge. Finally, the project did not assess actual return-to-driving times, objective driving ability, adherence to advice or driving-related adverse events. Improvements in documentation and awareness cannot therefore be assumed to translate into safer driving. 

Sustainability and future directions 

The principal intervention was simple, required no additional clinical equipment or separate educational programme, and was incorporated into an existing electronic workflow. These features may support sustainability, but persistence beyond the two-month follow-up remains untested. However, the project did not achieve the predefined 100% documentation target, with advice documented in 80.8% of post-intervention discharge summaries. Further work should examine why approximately one-fifth of summaries still lacked documentation. Where technically feasible, a mandatory or automated discharge prompt could further reduce omissions. More importantly, the next improvement cycle should place greater emphasis on comprehension while maintaining reliable information provision. Based on the present findings, a logical next PDSA cycle would retain the electronic template while introducing consistent PIL provision, brief verbal counselling, and structured junior doctor education. Patient awareness of DVLA requirements should remain a key outcome rather than relying solely on documentation compliance. 

Giannoudis et al. examined 592 patients following hip or knee arthroplasty and found that approximately 60% had resumed driving at six weeks. Driving status was associated with measures of functional recovery, including mobility, walking-aid use and pain [12]. This suggests that a fixed postoperative interval may not fully capture differences in recovery and supports considering functional readiness during counselling. Wilson et al. similarly identified variation in return to driving in a contemporary arthroplasty cohort, with 851 patients included in the analysis. Earlier return was associated with factors including surgical side, procedure type and surgical approach, as well as previous driving frequency [13].

These findings extend the discussion beyond elapsed time by highlighting the potential relevance of procedural factors and patients’ usual driving habits. Taken together, these observational studies support individualised counselling alongside general postoperative timeframes, but neither establishes that earlier resumption is safe. In the present project, a standardised template should therefore ensure consistent coverage of essential advice while allowing clinicians to tailor recommendations to individual recovery. Future improvement cycles could assess this individualisation alongside documentation completeness and patient comprehension. 

The predefined content of the driving-advice template may also inform future evaluation of AI-assisted discharge communication. In an exploratory study using three constructed discharge letters, Eisinger et al. found that GPT-4 improved the readability of discharge letters but omitted some patient safety information, highlighting the importance of preserving essential advice during automated drafting [14]. Rust et al. subsequently demonstrated improved patient activation (knowledge, skills and confidence in self-care) with clinician-reviewed, large language model-generated discharge summaries in a randomised controlled trial involving 128 medical inpatients [15]. Future QI work could therefore evaluate whether using structured electronic templates as a checklist during AI-assisted discharge summary drafting preserves essential advice while improving its clarity and patient comprehension. This potential application requires evaluation, as automation was not assessed in the present project.

## Conclusions

Embedding guideline-informed postoperative driving, DVLA, and insurance advice within the electronic discharge workflow was associated with a substantial improvement in documentation, from 0% to 80.8%. The composite of documented or patient-reported advice increased from 47.6% to 94.1%, driven by discharge-summary documentation; the analysis excluding this route did not demonstrate improved coverage through qualifying patient-reported routes. The intervention was a simple workflow change aimed at reducing reliance on individual clinician memory. Its sustainability remains unestablished and requires evaluation across subsequent staff rotations. Evidence of a change in DVLA awareness was inconclusive; fewer than half of post-intervention respondents met the survey criterion for awareness. Increased documentation did not establish that patients had received or understood the advice. Workflow standardisation appears effective for improving process reliability, but a multimodal approach incorporating patient-facing information, verbal reinforcement, and recurring clinician education may be required to improve comprehension among clinicians and patients alike. The next improvement cycle should therefore retain the electronic template while targeting the remaining gap between information delivery and understanding.

## Appendices

Appendix 1. Verbatim patient-survey questions

The telephone survey assessed patient-reported receipt of postoperative driving advice and awareness of DVLA notification requirements. At the start of each call, the data collector introduced themselves, confirmed the patient's identity, explained the purpose of the survey, and asked whether the patient agreed to participate. Participation was voluntary. Survey eligibility was then established before proceeding to questions about patient information leaflets, verbal advice, and DVLA awareness. The questions and assessment criteria are presented below.

Question 1: Survey Eligibility

“Do you currently drive or hold a valid driving licence?”

Patients meeting either criterion were eligible. The survey continued only with patients who agreed to participate.

Question 2: Patient Information Leaflet

“Did you receive a patient information leaflet containing advice about driving after your operation?”

This item assessed patient-reported receipt of a leaflet containing postoperative driving advice. Leaflet content was not independently evaluated.

Question 3: Receipt of Verbal Advice

“Did anyone give you verbal advice about returning to driving after your operation?”

This item assessed patient-reported receipt of verbal advice.

Question 4: Accuracy of Verbal Advice

For patients reporting receipt of verbal advice:

Open-ended question: “What advice were you given about returning to driving after your operation?”

Closed-ended follow-up: “Were you advised that you should be able to perform an emergency stop safely before returning to driving?”

Analysis: Verbal advice was classified as meeting the emergency-stop accuracy criterion if the patient recalled this advice in their initial response or confirmed having received it following the closed-ended question. This classification assessed patient-reported receipt of the specified advice, rather than independently verified counselling content or the completeness of the advice given.

Question 5: DVLA Awareness

Open-ended question: “What do you know about notifying the DVLA after surgery?”

Closed-ended follow-up: “Were you aware that, if your ability to start driving was affected for more than three months since your surgery, you would have to contact the DVLA?”

Analysis: DVLA awareness was recorded when patients either correctly identified the stated notification advice in their open-ended response or affirmed awareness during the closed-ended follow-up.

Appendix 2. Junior doctor questionnaire and scoring criteria 

The questionnaire comprised three multiple-choice questions, one confidence rating and one free-text question. Question wording and response options are reproduced as administered. 

Question 1

After a right total hip replacement, when can most patients safely return to driving? 

1-2 weeks 

2-4 weeks 

6 weeks 

3 months 

Unsure 

Scoring criteria: The proportion selecting “6 weeks” was reported. This response was consistent with the general timeframe described in the Royal College of Surgeons of England hip replacement leaflet, subject to individual functional recovery [4]. 

Question 2

After a right total knee replacement, typical timeframe to return to driving? 

2 weeks 

4-6 weeks 

8-12 weeks 

Unsure 

Scoring criteria: The proportion selecting “4-6 weeks” was reported descriptively. This response category did not precisely match the Royal College of Surgeons of England knee replacement leaflet, which suggests assessing fitness to drive after six weeks [5]. Selection of this category was therefore not interpreted as definitive evidence of guideline-concordant knowledge. 

*Question 3* 

Do patients legally need to notify the insurance agency/DVLA after a joint replacement? 

Yes, inform both after the surgery 

Not at all 

Inform the insurance agency straight after, but do not need to inform the DVLA 

Inform the insurance agency; only inform the DVLA if they have not been able to drive after three months 

Inform DVLA at 2 months; only inform insurance agency if there are complications to surgery 

Scoring criteria: The fourth option was the designated response for the questionnaire. The proportion selecting this option was reported. This item assessed recognition of the questionnaire’s combined insurance and DVLA advice [2,3]. The original question’s use of “legally” conflated insurer notification under policy terms with DVLA notification requirements. This ambiguity may have influenced interpretation and answer selection. Results therefore indicate selection of the designated response rather than a comprehensive or validated assessment of legal knowledge. The original wording is retained for transparency.

*Question 4* 

How confident are you at giving driving advice to post-total hip/shoulder/knee replacement patients? 

Response options: 1 (least confident), 2, 3, 4 or 5 (most confident)

Analysis: Confidence scores were summarised using the mean and median (range), with higher scores interpreted as greater confidence. No correct or incorrect response was assigned. 

Question 5

What key advice should we give to post-arthroplasty patients to ensure safe return to driving? (type unsure if you are not sure) 

Response format: Free text. 

Analysis: Responses were classified according to whether they explicitly mentioned the ability to perform an emergency stop. Responses containing this criterion were counted even if they also expressed uncertainty. Responses consisting only of “Unsure” or mentioning other considerations without an emergency-stop criterion were not counted as identifying this criterion. This measure assessed recognition of one functional criterion rather than the completeness of the advice provided.
